# Structure and electrochemical performance of electrospun-ordered porous carbon/graphene composite nanofibers

**DOI:** 10.3762/bjnano.11.112

**Published:** 2020-08-27

**Authors:** Yi Wang, Yanhua Song, Chengwei Ye, Lan Xu

**Affiliations:** 1 National Engineering Laboratory for Modern Silk, College of Textile and Engineering, Soochow University, 199 Ren-ai Road, Suzhou 215123, China

**Keywords:** carbon/graphene composite nanofibers, carbonization, electrochemistry, electrode material, electrospinning method, ordered and porous nanofibers, supercapacitor

## Abstract

Ordered carbon/graphene composite nanofibers (CGCNFs) with different porous configurations were used as a material to fabricate supercapacitor electrodes. These nanofibers were synthesized by applying a modified parallel electrode to the electrospinning method (MPEM) in order to generate electrospun polyacrylonitrile (PAN) nanofibers containing graphene. After synthesis, these fibers were submitted to carbonization under a N_2_ atmosphere at 1100 °C. The influence of the ordering and porosity of CGCNFs on their electrochemical performance was studied. The results showed that by adding deionized water to the spinning solution one could increase the number of mesopores and the specific surface area of CGCNFs, thereby significantly increasing their specific capacitance. In addition, the ordering of CGCNFs within the electrode improved the electron transfer efficiency, resulting in a higher specific capacitance.

## Introduction

As the technology sector develops, societal demands for energy storage devices also increases. Supercapacitors, including electric double-layer capacitors (EDLCs) and pseudo-capacitance devices [1–2], are one of the most needed energy storage devices. Their main characteristics include high energy density, high power density, and fast charging speed [3–5]. These instruments have electrodes that are composed of either carbonaceous materials (carbon nanotubes, graphene, carbon nanofibers) or metal oxides (manganese oxide, nickel oxide, RuO_2_, Co_3_O_4_, etc.). Carbon is the primary material used to manufacture EDLC electrodes since it has a high specific surface area, which can easily form a double layer to store more electrical energy [6–10]. Since there is still room for improvement of the current supercapacitor electrochemical performance, the capacitance and cycle stability of supercapacitors are still subjects of research interest.

Electrospinning is one of the most convenient methods to synthesize nanofibers in a continuous manner. Electrospinning has many advantages over other methods in that it is simple, highly reliable, and not expensive. Due to the fact that the electrospinning solution can be easily modified, electrospun nanofibers, with different structures and properties, can be prepared by dissolving and mixing different substances [11–12]. Electrospun nanofibers have been widely used as a material to synthesize electrodes upon a carbonization step [13–14]. Polyacrylonitrile (PAN) is often used as a precursor to synthesize carbon nanofibers. It can be obtained from a variety of sources and it has good spinnability [14–15]. However, carbon-based materials generally have a stable and uniform internal structure, which results in a low specific surface area [16]. Pore formation is a strategy that has been used to increase the specific surface area of these materials. The porous structure not only increases the specific surface area but also facilitates ion transport. There are various ways to induce pore formation, including the activation and template methods [17–19]. In the context of electrospinning experiments, an effective method to induce porous formation is to change the composition of the electrospinning solution (e.g., by adding polymers that are incompatible with PAN, such as polystyrene (PS), poly(ʟ-lactic acid) (PLLA) and poly(methyl methacrylate) (PMMA) [20–23]. During the carbonization process at a high temperature, PAN and the blended polymers undergo phase separation, forming a large number of pores which increases the specific surface area. The increase in the specific surface area of the electrode due to increased porosity facilitates ion transportation, which increases the conductivity of monolithic electrodes [24–26].

Although the porous carbon nanofibers have a high specific surface area, their low electrical conductivity impedes their use in high-power-density supercapacitors. Therefore, by adding high-performance conductive materials one can enhance the electrochemical performance of carbon nanofibers. Experiments have shown that by introducing graphene into the carbon matrix, various mechanical and electrochemical properties of the original carbon matrix can be significantly improved [27]. The perfect crystalline graphene has an ideal two-dimensional crystal structure composed of a stable regular hexagonal lattice, which has an excellent theoretical specific surface area, increased electron mobility, high electrical conductivity and good biocompatibility [28–30]. Studies have indicated that graphene still maintains an excellent charge/discharge performance at an electrochemical scan rate of almost 250 mV·s^−1^ [31] and has an excellent cycle performance and fast charge/discharge characteristics [32].

Generally, the structures of nanocomposites used in electrochemical supercapacitors can influence their capacitance, charge and discharge rates, as well as their cycle stability. In our previous work [33], the ordered porous PAN/graphene composite nanofibers (OPPGCNFs) were prepared by a modified parallel electrode electrospinning method (MPEM). It was found that the alignment of the composite nanofibers (CNFs) improved their electrical conductivity. Therefore, this study provided a convenient and straightforward approach to synthesize ordered porous carbon/graphene CNFs (CGCNFs) with a high number of mesopores to be used as a material to synthesize supercapacitor electrodes. The method used in our previous work to induce porosity was the carbonization of the OPPGCNFs, obtained by MPEM, at 1100 °C under a N_2_ atmosphere [33]. The results showed that the number of mesopores and the specific surface area of the fabricated CGCNFs were enhanced by adding deionized water (DIW) to the spinning solution. The alignment and the increased number of mesopores in the CGCNFs significantly enhanced the electrochemical performance of these electrodes, which was corroborated by an increase in the specific capacitance of CGCNFs (from 35.65 F·g^−1^ to 151.34 F·g^−1^) when MPEM was used and DIW was added to the spinning solution.

## Experimental

### Materials

PAN (*M*_w_ = 150,000) and *N*,*N*-dimethylformamide (DMF) were purchased from Aldrich Chemical Co. Ltd (USA). Graphene was purchased from Sigma-Aldrich Trading Co. Ltd (Shanghai, China). DIW was prepared in the laboratory. All materials were used as received without any further purification.

### Preparation of electrospinning solutions

The electrospinning solution was prepared by dispersing PAN, graphene and DIW in DMF. First, 12 wt % of PAN was dissolved in DMF and magnetically stirred at room temperature for 2 h until a homogeneous solution was obtained. Then 0.5 wt % of graphene was added to the PAN/DMF solution, which was submitted to ultrasonic vibration for 30 min until graphene was well-dispersed in the solution. Finally, DIW was added to the graphene/PAN/DMF solution upon magnetic stirring for 2 h to obtain a 2 wt % homogeneous electrospinning solution.

### Preparation of carbon/graphene composite nanofibers

**Figure 1:**
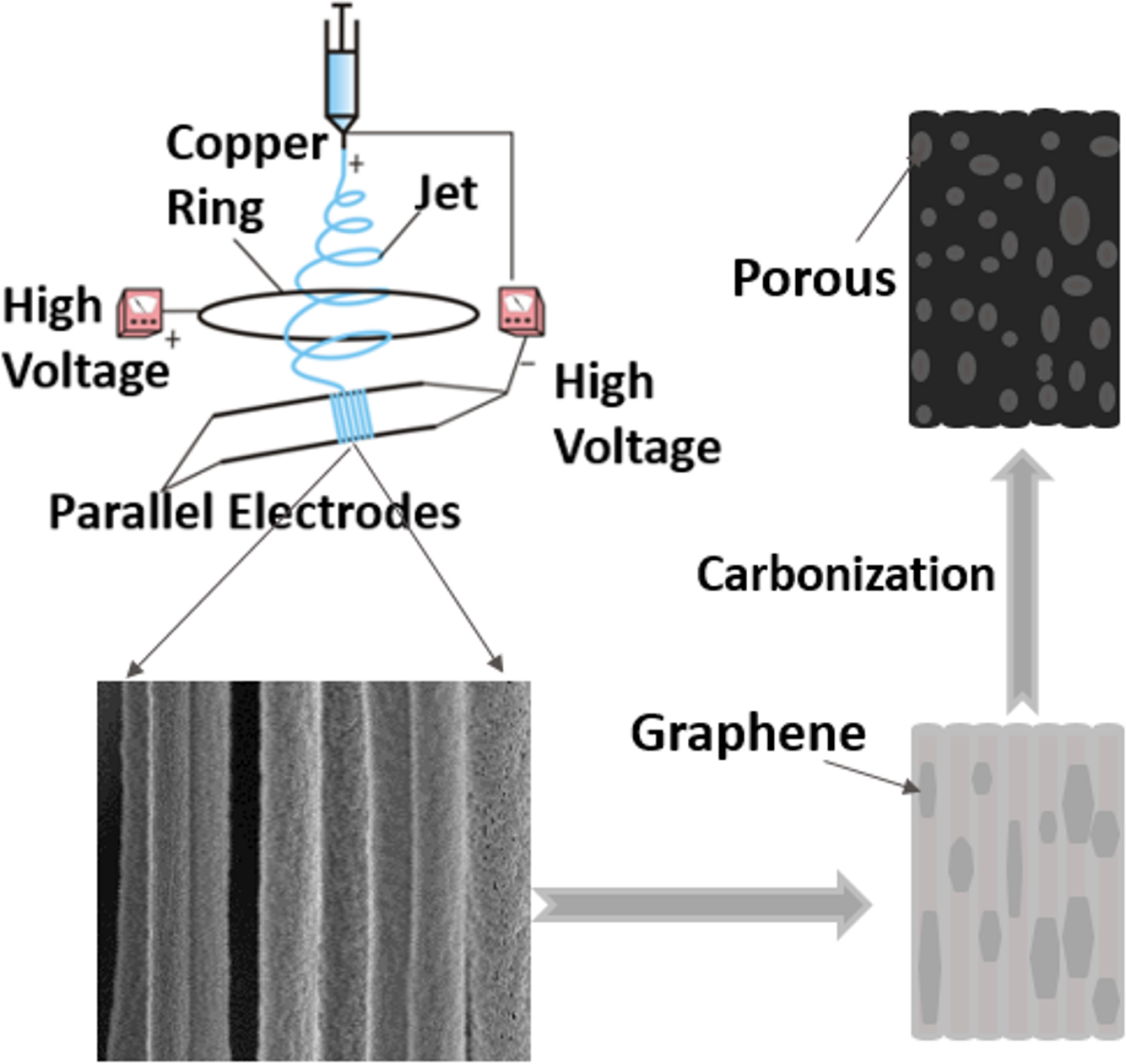
Diagram of the preparation process of the ordered porous CGCNFs. Setup scheme adapted from [33], distributed under the terms and conditions of the Creative Commons Attribution (CC BY) license (http://creativecommons.org/licenses/by/4.0/).

The ordered porous CGCNFs were prepared by MPEM and then submitted to carbonization under a N_2_ atmosphere at 1100 °C, as shown in Figure 1. The MPEM apparatus is composed of two high-voltage power generators (NTPS-35K, Ntsse Co., Korea), a flow pump (LSP01, Longerpump Co., Ltd., China), a syringe (20 mL) with a capillary tip (diameter = 0.5 mm), a copper ring and a parallel electrode collector. The needle tip and the copper ring were clamped on the anode of the high-voltage power supply, whereas the parallel electrodes were connected to the cathode [24].

According to our previous work [33], electrospun-ordered PAN/graphene CNFs (PGCNFs) were prepared by MPEM at room temperature and 50 ± 5% relative humidity. The spinning solution was placed in the syringe and delivered by the flow pump at a 1 mL·h^−1^ flow rate. The applied spinning voltage was 18 kV, the spinning distance was kept at 18 cm, the distance between two paralleled electrodes was 5 cm, the diameter of ring was 21 cm, and the voltage applied to the ring was 5 kV.

Then the PGCNF membranes were cut into a square shape (e.g., 3 cm × 3 cm), put into a muffle furnace (KSY-D-16, Longkou Precision Instrument Co., Ltd., China), and submitted to a preoxidation step at 280 °C for 8 h under air at a heating rate of 1 °C·min^−1^. The high temperature during the oxidation process induced phase separation between PAN and DIW and triggered the initiation of cross-linking and cyclization of PAN. Finally, the preoxidation product was further carbonized at 1100 °C for 2 h in a tube furnace (OTF-1200X-II, Hefei Kejing Material Technology Co., Ltd., China) under a N_2_ atmosphere at a heating rate of 5 °C·min^−1^. Afterwards, the tube furnace was cooled to room temperature to obtain CCGNFs, which could be directly used as electrodes. For comparison, disordered CGCNFs were also prepared using electrospinning and carbonization techniques.

### Material characterization

The morphology of the CNFs was investigated via scanning electron microscopy (SEM) under a field-emission scanning electron microscope (FE-SEM) (Hitachi, S4800, Japan). The structural characteristics of the CNFs were observed via transmission electron microscopy (TEM) (FEI, Tecnai G20, Japan). The diameter values of the CNFs were measured using ImageJ software (National Institute of Mental Health, USA). The Brunauer–Emmett–Teller (BET) specific surface area and porosity of the CCGNFs were determined by using a surface area analyzer (Micromeritics, ASAP 2020, USA) at 77 K, taking into consideration the N_2_ adsorption and desorption isotherms. X-ray diffraction (XRD) analysis (Rigaku D/Max-rB, Japan), with diffraction angle values ranging from 5° to 60°, was performed to examine the crystalline structure of CCGNFs. A Fourier-transform infrared spectrometer (FTIR) (Frontier, Perkin-Elmer Company, USA) was used to investigate the structural changes of the CNFs before and after carbonization and the reaction between the polymer and graphene.

### Electrochemical evaluation and capacitive deionization experiments

The CGCNF membranes were cut into small pieces (1.0 cm × 1.0 cm) and oven- dried (DZF-6050, Shanghai Jinghong Scientific Instrument Co. Ltd., China) for one hour at 50 °C. Then the dried CGCNF membranes were weighed to an accuracy of 0.001 g and clamped to a platinum plate electrode holder. After clamping, the CGCNF membranes were directly used as cell electrodes and immersed into an electrolyte solution. The electrochemical measurements were performed in a standard three-electrode cell at room temperature. A graphite rod was used as the counter electrode, Hg/HgO was used as the reference electrode, and a 6.0 M KOH aqueous solution was used as the electrolyte solution. The electrochemical performance of the CCGNFs was investigated using an electrochemical station (CHI660E, Chenhua, Shanghai) by using cyclic voltammetry (CV), electrochemical impedance spectroscopy (EIS), and constant current galvanostatic charge/discharge (GCD) techniques. A voltage range of 0–1.0 V was applied to the CV electrode at a scan rate of 25 mV·s^−1^. The impedance measurements of the electrode alternating current (AC) were performed at frequencies ranging from 0.01 Hz to 100 kHz.

## Results and Discussion

**Figure 2:**
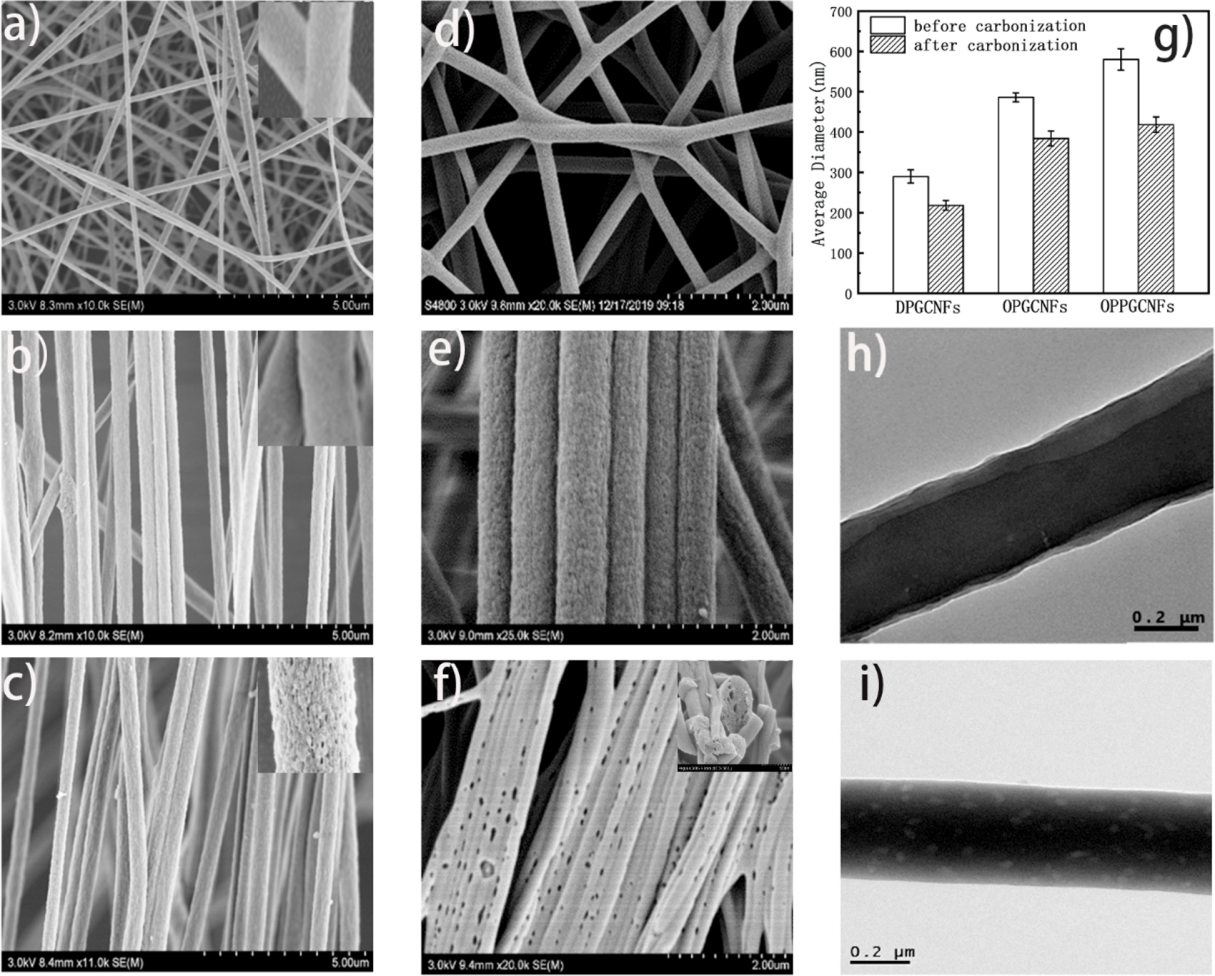
FE-SEM images of (a) disordered PGCNFs synthesized via electrospinning (DPGCNFs) with an average diameter of 289.9 ± 16.5 nm. (b) Ordered PGCNFs synthesized via MPEM (OPGCNFs), with an average diameter of 486.2 ± 11.1 nm. (c) Ordered porous PGCNFs synthesized via MPEM (OPPGCNFs), with an average diameter of 579.9 ± 26.6 nm. (d) Disordered CGCNFs (DCGCNFs), with an average diameter of 218.2 ± 12.2 nm. (e) Ordered CGCNFs (OCGCNFs), with an average diameter of 384.1 ± 18.5 nm. (f) Ordered porous CGCNFs (OPCGCNFs), with an average diameter of 418.4 ± 19.1 nm. (g) Average CNF nanofiber diameter values before and after carbonization. TEM pictures of PGCNFs (h) and CGCNFs (i).

The surface and cross-section morphologies of the CNFs before and after carbonization were examined via SEM and TEM, respectively, as shown in Figure 2. According to Figure 2a, the PGCNFs prepared via electrospinning were disordered, while the PGCNFs synthesized via MPEM were highly ordered (Figure 2b and Figure 2c). The explanation for these results lies in the fact that after the copper ring was added, the number of electric charges on the surface of the stretched jet increased. As a consequence, the resultant force generated by the copper ring also increased, leading to an increase in the jet kinetic energy and acceleration of the jet downward stretching speed [34]. Therefore, the results showed that MPEM improved both the stability and ordering of electrospun nanofibers, while reducing the nanofiber diameter values. In addition, due to the volatility of DIW and DMF, visible pores appeared on the surface of PGCNFs after DIW was added to the spinning solution, as indicated in Figure 2c. The PGCNFs with different morphologies were named DPGCNFs (disordered PGCNFs by electrospinning), OPGCNFs (ordered PGCNFs by MPEM) and OPPGCNFs (ordered porous PGCNFs by MPEM) respectively.

The morphologies of the carbonized CNFs, called CGCNFs, were also illustrated in Figure 2d-f. CGCNFs with different morphologies were named DCGCNFs (carbonized DPGCNFs), OCGCNFs (carbonized OPGCNFs) and OPCGCNFs (carbonized OPPGCNFs). Compared with the PGCNFs before carbonization, CGCNFs retained the initial ordering. The pores in the CGCNFs appeared due to the cross-linking and cyclization of PAN during carbonization. In addition, other pores in the OPCGCNFs, in a larger amount and size, were generated either by carbonization of the electrospun OPPGCNFs or by the addition of DIW during the spinning process. In fact, DIW was not fully evaporated during the spinning process. The rest of the DIW in the OPPGCNFs was evaporated by further increasing the temperature during the carbonization process. Many pores were formed not only at the surface of the CCGNFs but also inside these fibers. The pore formation not only increased the specific surface area of the CGCNFs but also had a significant influence on the ionic conduction in the electrolyte solution, thereby affecting the final electrochemical performance of the CGCNFs. Figure 2g showed the average diameter values of CNFs before and after carbonization. As expected, the average diameter values of ordered CNFs, synthesized via MPEM, were higher than the diameter values of disordered CNFs obtained via electrospinning. This is due to the suppressed bending instability of jets in the MPEM process, leading to higher average diameter values for ordered-porous CNFs in comparison to ordered nonporous CNFs, as a result of pore generation. In addition, the CNF average diameter values before carbonization were all higher than the values measured in CNFs after carbonization due to the combined effect of thermal drafting and chemical reaction. Given that there is an uneven heat transfer and diffusion during the carbonization process, the chemical structure of the CNFs is distributed along a radial gradient, resulting in a thermal drafting effect. As the temperature rises during the carbonization process, differences in the radial chemical structure of the CNF gradually increase, resulting in an increase of the internal tension. The tension regulates the shape and structural reorganization of the crystallites in the CNF, leading to a smaller CNF diameter.

The distribution of graphene in PCGNFs and CCGNFs was determined via TEM (Figure 2h and Figure 2i, respectively). The results show that graphene was successfully introduced in the CNFs and arranged in an orderly manner along the CNF axes. Moreover, the CGCNF TEM micrograph indicated that there was an apparent porous structure on the surface of the CGCNFs, due to the decomposition of the PGCNFs and PAN coating during carbonization.

### FTIR and XRD spectra analysis

**Figure 3:**
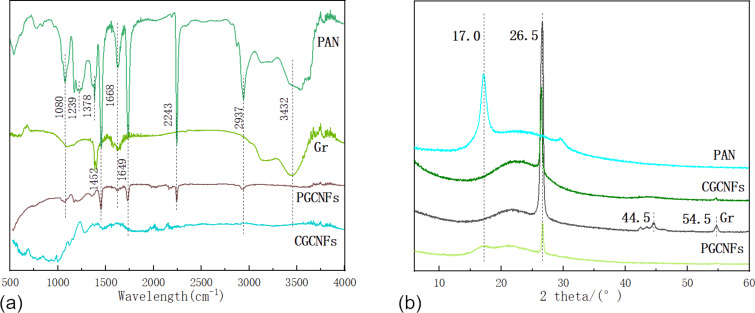
(a) FTIR spectra of PAN nanofibers (dark green), graphene (light green), PGCNFs (purple) and CGCNFs (cyan). (b) XRD results of PAN nanofibers (cyan), graphene (gray), PGCNFs (light green) and CGCNFs (dark green).

The FTIR spectra were used to analyze whether there was any interaction between PAN and graphene. In the PAN spectrum, the absorption peaks at 1239, 1378 and 1452 cm^−1^ were due to the bending of the C−H of PAN, the absorption peak at the wavelength of 2243 cm^−1^ was attributed to the stretching vibration of C−N, and the absorption peak at 1668 cm^−1^ was generated by the stretching vibration of C=C [35]. As shown in Figure 3a, upon addition of graphene, the spectra of PAN nanofibers exhibited a few minor changes. One of these changes was in the region of 3300–3500 cm^−1^, due to the interaction between graphene electrons and the hydrogen attached to the nitrogen atoms in the carbamate bond, changing the shape of the absorption peak. After carbonization, the absorption peak at 2243 cm^−1^ disappeared. This peak was related to a cyano group of PAN and its disappearance indicated PAN decomposition after carbonization. The absorption peaks at 1000–1500 cm^−1^ were the characteristic peaks of the carbon skeleton. However, their intensities were minimal, indicating that most of the CNFs were decomposed after carbonization, leaving behind only the carbon skeleton in the CGCNFs.

Figure 3b shows PAN nanofibers, graphene, PGCNFs and CGCNFs crystalline structures obtained via XRD. According to the XRD results, the peak observed at 2θ = 17° can be designated as the (200) PAN crystal plane [30]. The peaks at approximately 26.5°, 44.5°, and 54.5° in the XRD spectra were typical peaks of either graphite or graphene [36–37]. These peaks were related to the (002) diffraction plane of hexagonal graphite structures in carbon materials. The XRD patterns of PAN/graphene all contained PAN and graphene characteristic peaks and no new peak was identified in the PGCNFs, suggesting that there was no chemical reaction happening between PAN and graphene. The characteristic peak at 26.5° was present before and after carbonization; however, the characteristic peak at 17.0° disappeared after carbonization due to the decomposition of PAN during the process. Moreover, the diffraction peak intensity of the (002) crystal plane of the carbonized graphite structure was significantly enhanced, indicating that the carbonization process enhanced the crystallinity of the graphite composite, which was also a consequence of the increase in the amount of graphite in the composite.

### Nitrogen sorption analysis

**Figure 4:**
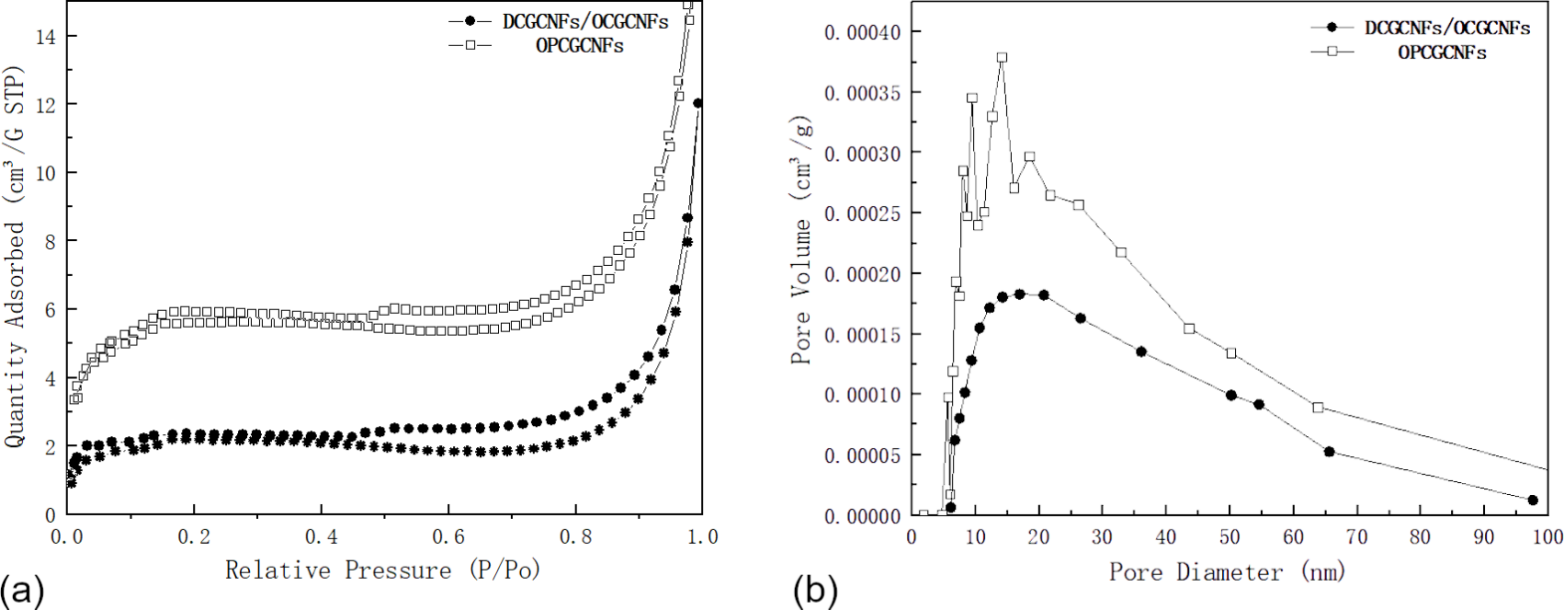
(a) Nitrogen adsorption–desorption isotherms and (b) their corresponding PSD curves determined by the BJH method applied to DCGCNFs/OCGCNFs and OPCGCNFs.

The pore structure characteristics and specific surface area of CGCNFs were determined by N_2_ adsorption/desorption isotherms at 77 K. Nitrogen adsorption/desorption isotherms and their corresponding pore-size distribution (PSD) curves were obtained by using Barrett–Joyner–Halenda (BJH) analysis, as illustrated in Figure 4. The adsorption isotherms of DCGCNFs, OCGCNFs, and OPCGCNFs in Figure 4a showed a typical type IV behavior. There was a visible hysteresis loop between the adsorption and desorption nitrogen branches. In addition, the hysteresis loops in the *P*/*P*_0_ 0.5–1.0 range demonstrated that the material had a significant mesoporous structure [38]. These porous structures are able to provide lower resistance channels and shorter transfer paths for ions in the electrolyte. This is due to the fact that large mesoporous holes are more suitable for rapid ion diffusion at a high-load current density. This way, the specific surface area generated by these pores is effectively utilized [39]. At relatively high pressure values (*P*/*P*_0_ > 0.5), there was an evident hysteresis loop due to capillary condensation in the mesopores, which also indicated a high mesoporous content [40]. At relatively low pressure levels (*P*/*P*_0_ < 0.1), the amount of nitrogen adsorbed on the sample was almost insignificant, which indicated the existence of negligible micropores. Moreover, the increased nitrogen adsorption at a high relative pressure (*P*/*P*_0_ > 0.8) illustrated that the sample had a certain amount of macropores, which was beneficial to the kinetic process during the contact between the electrolyte and the electrodes, providing a better electrolyte-buffer base [41]. The PSD curves shown in Figure 4b suggested that the average pore diameter values of DCGCNFs/OCGCNFs and OPCGCNFs were 37.24 nm and 30.89 nm, respectively. The pore diameter values of all samples were mainly distributed over the range of 5–65 nm, which was consistent with the adsorption isotherm results, indicating that mesopores accounted for the majority of the porous structures, although there were also some macropores. The existence of macropores was probably due to the fact that graphene and DIW were not evenly dispersed within the samples, leading to the formation of excessively large pores during carbonization.

Compared with DCGCNFs/OCGCNFs, the adsorption volume of OPCGCNFs was gradually increased, indicating that porosity also increased. It was evident that DIW became a pore-making agent in the high-temperature carbonization process. Upon the addition of DIW, heating and DIW evaporation, many mesoporous structures were formed in the OPCGCNFs. These structures had a maximum specific surface area of 17.5889 m^2^·g^−1^, which shows that the addition of DIW effectively increased the pore volume and surface area of the sample. The results were in accordance with the SEM images of the CGCNFs, which showed even more interconnected mesoporous channels in the inner and outer surfaces of OPCGCNFs compared with DCGCNFs/OCGCNFs. The high amount of mesoporous channels was beneficial to the high-speed ion transport and adsorption. The PSD curves were used to analyze, in more detail, the differences in pore structure between different samples. The surface area and pore parameters of all samples are summarized in Table 1. The specific surface area, average pore diameter per total pore volume, and average pore diameter of DCGCNFs/OCGCNFs were 6.89 m^2^·g^−1^, 0.017 cm^3^·g^−1^, and 37.24 nm, respectively. In contrast, the corresponding values for OPCGCNFs were 17.59 m^2^·g^−1^, 0.027 cm^3^·g^−1^, and 30.89 nm, respectively.

**Table 1:** Pore characteristics of DCGCNFs/OCGCNFs and OPCGCNFs.

Samples	*S*_BET_^a^ (m^2^ g^−1^)	*V*_t_^b^ (cm^3^ g^−1^)	Pore volume fraction (%)		APD^c^ (nm)

			Micropore	Mesopore	

DCGCNFs/OCGCNFs	6.89	0.017	75.60%	24.40%	37.24
OPCGCNFs	17.59	0.027	71.19%	28.81%	30.89

^a^The specific surface area (*S*_BET_) was calculated using the BET method. ^b^*V*_t_ represents the total pore volume. ^c^APD indicates the average pore diameter calculated using the BET method.

As shown in Figure 4 and Table 1, both the specific surface area and mesoporous volume of the electrode were significantly enhanced by adding DIW. From the corresponding SEM and TEM images it can be inferred that the increase in the pore volume of the OPCGCNFs was mainly due to the increase in the number of channels in the fibers, leading to an increase in the ion transport rate. This shows that proper microporous/mesoporous structures can facilitate the transport and adsorption of ions in the electrode, which favors the electrochemical process [42].

### Electrochemical characterization

**Figure 5:**
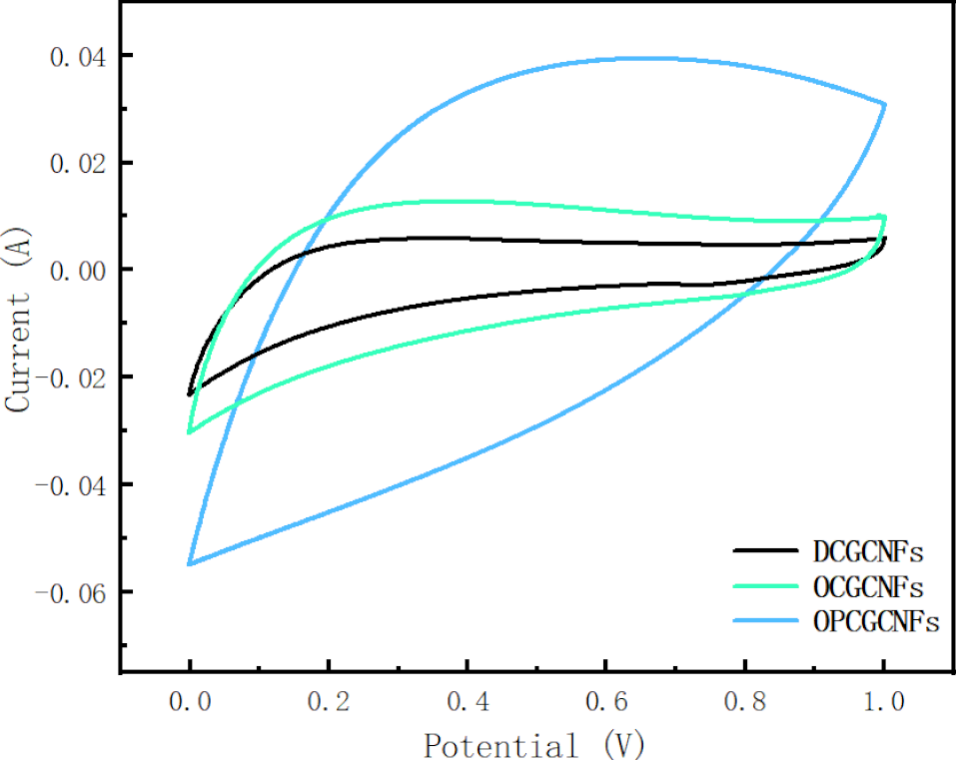
CV curves of the CGCNF electrodes at a scan rate of 25 mV/s.

Cyclic voltammetry is a reliable method to analyze the capacitance of supercapacitor electrodes [43]. Capacitors made of CGCNFs were submitted to a CV experiment at a scan rate of 25 mV·s^−1^ and the obtained curves are shown in Figure 5. All the CV curves were box-shaped, indicating that no visible redox peaks were observed during the reversible electrochemical process. The results also confirmed that all the samples had good electric double-layer capacitance over a range of 0–1.0 V [44]. Moreover, the OPCGCNF electrode had the most extensive induced current and the largest box-like shaped curve, demonstrating that the OPCGCNF electrode had the largest capacitance among the three CGCNF electrodes analyzed and had a very rapid charging/discharging reaction [45]. The high capacitance of the OPCGCNF electrode could be attributed to its higher mesopore volume fraction (Figure 4 and Table 1). The mesoporous structures provided a shorter path and a lower resistance for ion diffusion in porous electrodes by increasing the electrode specific surface area. Therefore, mesoporous structures are more suitable for high-speed ion diffusion under high-load current density [27]. In addition, ordered CCGNF electrodes had higher capacitance in comparison to disordered CCGNF electrodes since the ordered structure of CCGNFs was more favorable to the rapid ion diffusion in low-resistance paths [46].

The specific capacitance of the electrodes was calculated from the CV curve by using the following equation [47–48]:


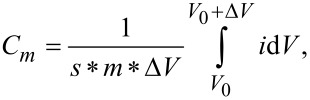


where *m* is the mass of the electrode (g), *s* is the potential scan rate (mV/s), *i* is the response current (A) and Δ*V* is the potential window (V, Δ*V* = 1.0).

The calculated specific capacitance values of the electrode were 35.65 F·g^−1^ for DCGCNFs, 90.68 F·g^−1^ for OCGCNFs, and 151.34 F·g^−1^ for OPCGCNFs, considering a 6 M KOH electrolyte solution. The results showed that the OPCGCNF electrode had the highest SC values compared to the other electrodes (OCGCNF and DCGCNFs). This result shows that the material porosity plays a significant role in improving the capacitive properties of a given electrode. According to the diffusion kinetics of ions in solution, the high specific surface area and high porosity of the electrode material allow for a quick ion adsorption on the electrode surface, thereby improving the migration rate of ions and reducing the charge diffusion resistance. In addition, the specific capacitance values of OCGCNFs were higher than the values obtained for DCGCNFs, since the ordered structure of the CGCNF electrodes significantly enhanced the electrochemical performance of the electrodes. The ions in the electrolyte diffused and were transferred in a particular order, enhancing the ion transmission efficiency and the electrode electrochemical performance.

**Figure 6:**
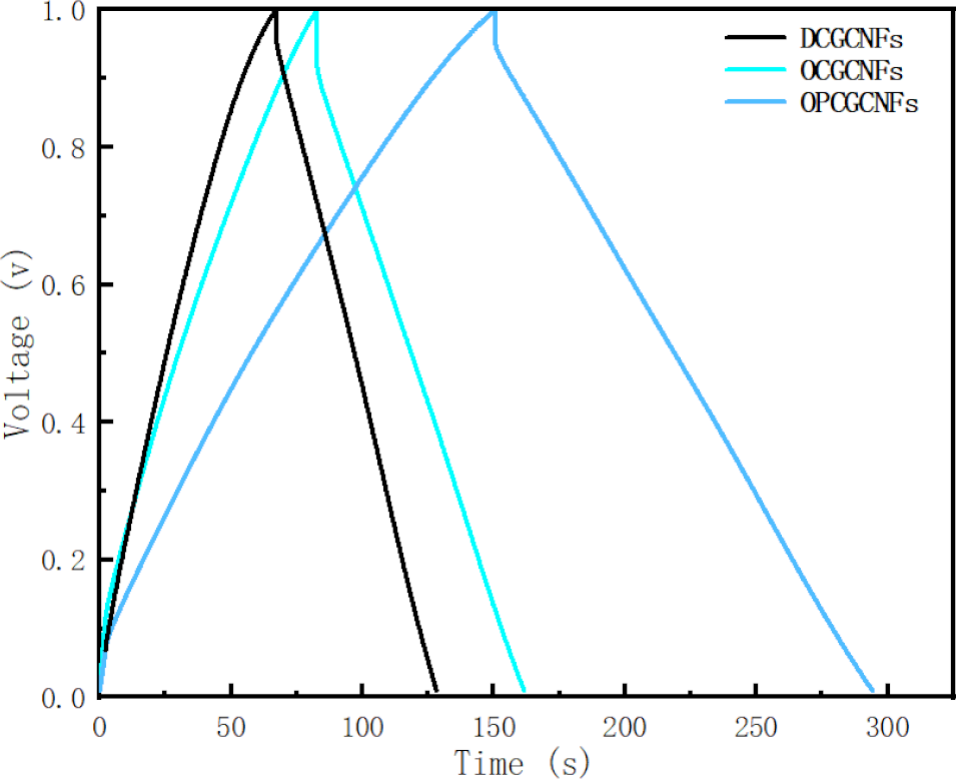
Charge/discharge curves of samples in a 6 M KOH electrolyte solution.

Figure 6 shows the charge/discharge curve of a CCGNF electrode in a 6 M KOH solution at a constant current (1.0 A·g^−1^) and voltage (1.0 V). The increasing order of discharge time was DCGCNFs < OCGCNFs < OPCGCNFs. The OPCGCNF electrode, with an ordered fiber structure and more mesopores, showed the longest discharge time due to the largest specific surface area and highest capacitance. The most significant results were the charge and discharge time values between the OCGCNFs (161 s) and OPCGCNFs (294 s) (time difference of approximately 133 s). These results can also be explained by the increase in specific surface area and capacitance. In addition, due to the ordered fibrous structure in the electrode, the charge and discharge time difference between DCGCNFs (128 s) and OCGCNFs (161 s) was 33 s. From a microscopic point of view, the ordered fibrous structure of the electrode allows for electrons to transfer and diffuse more quickly to a certain extent, thereby improving their charge and discharge efficiency. In addition, the galvanostatic charge/discharge curves of these electrodes exhibited similar symmetrical isosceles triangles, which were consistent with the characteristics of the double-layer capacitor electrode and with the results of the CV curves.

**Figure 7:**
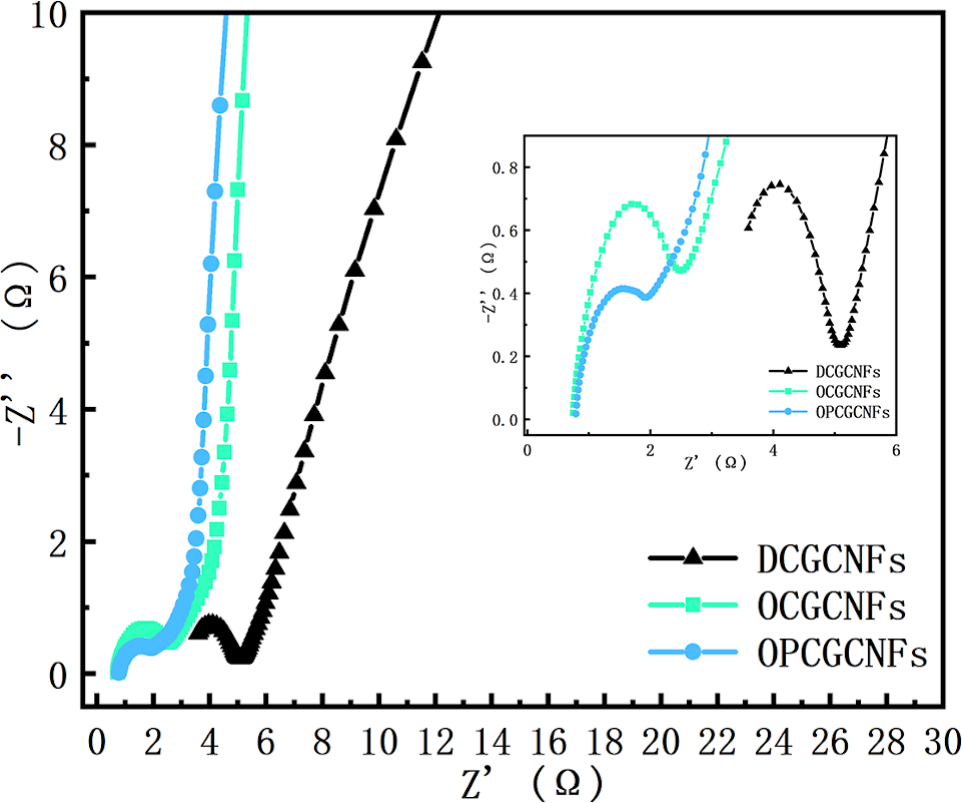
Electrochemical impedance spectra of the samples represented as Nyquist plots.

Electrochemical impedance spectroscopy (EIS) is a reliable method used to characterize electrode electrochemical behavior [49]. EIS is one of the most accurate methods to analyze the dynamic process of diffusion in the electric double layer of an electrode. It is also commonly used to study the high energy storage capacity mechanism in electrodes. The general EIS spectrum is mainly composed of two parts: the high frequency region and the low frequency region. The high-frequency region often exhibits a semicircular shape, and the arc of this section reflects the characteristics of the microscale interface between the electrolyte and the electrode [35]. The linear part of the low-frequency region generally represents the material transfer resistance, which corresponds to the diffusion-limiting process. The approximate capacitance of the capacitor is calculated by using the low-frequency data. In this work, EIS measurements were conducted in a frequency range varying from 0.01 Hz to 100 kHz. The internal resistances of DCGCNF, OCGCNF and OPCGCNF electrodes were 3.2, 0.8 and 0.7 ohm, respectively, as shown in Figure 7.

The diameter of the semicircle in the high-frequency region is a direct representation of the charge transfer resistance (*R*_ct_). Therefore, the smaller the semicircle diameter, the smaller the charge transfer resistance. The charge transfer resistance is related to both the conductivity of the electrode and the morphology of the active material (i.e., surface area and pore size, respectively) [50–51]. The *R*_ct_ values for DCGCNF, OCGCNF and OPCGCNF electrodes were 1.6, 1.2, and 1.0 ohm, respectively. The charge transfer resistance of the OPCGCNF electrode was significantly smaller than of the other CGCNF electrodes, as illustrated in Figure 7.

The low-frequency curve is related to the diffusion resistance of the electrolyte and ions into the electrode [52–53]. The almost vertical shape represents the rapid ion diffusion in the electrolyte and the adsorption on the surface of the electrode, indicating the ideal capacitance behavior of the electrode. In addition, the more vertical the curve is in the low-frequency region, the more ideal the supercapacitor [42–43]. Comparing the impedance curves of these electrodes, the slope of the curve corresponding to the OPCGCNF electrode was higher in the low-frequency region, as displayed in Figure 7, indicating that it was more favorable to the diffusion of k^+^ from the electrolyte to the surface of the electrode [9].

These results demonstrated that OPCGCNFs can be used as electrodes due to their advantages, such as fast ion dynamics, high electron conductivity and low electron transport impedance. This may be due to the following two factors: (i) The ordered fiber structure of the OPCGCNF electrode allows for the ions to move in an orderly manner in the electrolyte, improving the electron transfer efficiency while reducing the charge transfer resistance. (ii) By applying a pore-forming agent to OPCGCNF the electrode specific surface area increases and acquires many mesopores, which significantly facilitates the transport of electrons. In addition, a large number of mesopores in the carbon material potentially improves the diffusion kinetics of ions in both electrolyte and electrodes, enhancing the electrolyte transfer rate to the electrode pores while maintaining high capacitance retention [54]. These two factors explain why decreasing the diffusion distance to the nanometer range not only promoted charge transfer but also reduced internal resistance. Moreover, the reduction in resistance increased the current density on the electrode surface, increasing the diffusion rate in which ions are transferred from the electrolyte to the electrode. In summary, the OPCGCNFs with a larger number of mesopores had an improved electrochemical performance.

## Conclusion

The ordered CGCNFs with different porous configurations were used here as a material to manufacture electrodes for supercapacitors. These electrodes were fabricated by MPEM from the PAN/DMF spinning solution containing graphene, followed by a carbonization process under a N_2_ atmosphere at 1100 °C. The electrochemical performance of the supercapacitor electrode was also investigated. The results showed that the ordered and porous structures of the CGCNFs significantly impacted the CGCNF electrochemical performance. By improving fiber alignment, increasing the number of mesopores and enhancing the electrode specific surface area, one can effectively improve the electrochemical performance of an electrode. These improvements can significantly contribute to the electronic and ionic transport by decreasing the transfer resistance of electrodes.

When compared to DCGCNFs and OCGCNFs, OPCGCNFs have a highly ordered structure and a larger number of mesopores. These features were achieved by simply using MPEM and adding DIW in the spinning solution. In addition, OPCGCNFs had a better electrochemical performance, higher specific capacitance (151.34 F g^−1^ compared to 35.65 F g^−1^, which is the capacitance of CGCNFs), longer discharge time and smaller charge-transfer resistance. Therefore, OPCGCNFs can be used as a material to fabricate supercapacitor electrodes in future applications
